# *S*‑Palmitoylation in Health and Disease

**DOI:** 10.1021/acs.biochem.6c00247

**Published:** 2026-06-05

**Authors:** WanTing Wong, Mingyang Hu, Rivka L. Isaacson

**Affiliations:** Department of Chemistry, 4616 King’s College London, Britannia House, 7 Trinity Street, London SE1 1DB, U.K.

**Keywords:** *S*-palmitoylation, post-translational modification, protein localization, signal regulation, disease pathogenesis, thioester bond

## Abstract

*S*-palmitoylation is a reversible protein post-translational modification whereby a 16C fatty acid chain is attached to a cysteine residue via a thioester bond. This modification is crucial in regulating protein localization, conformation, stability, and interaction with other molecules. It influences multifarious physiological functions, from immune signaling to cellular apoptosis. In recent years, protein palmitoylation and diverse disease pathogenesis have been increasingly linked; this review intends to present a recent overview of the area, covering its catalysis mechanisms, functional significance, and role in diseases while discussing research challenges to strengthen our understanding of *S*-palmitoylation. Rather than providing an exhaustive summary, this review focuses on specific recent exemplars to illustrate the biological importance of *S*-acylation.

## Graphical abstract

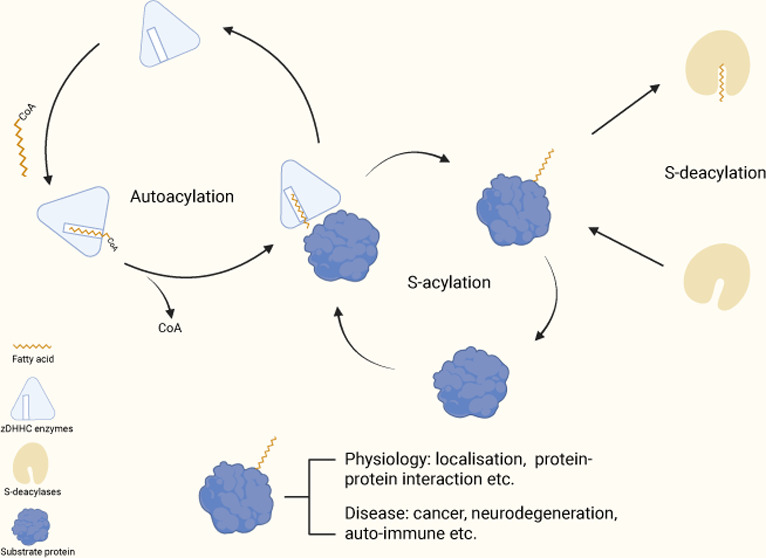

## Introduction

Proteins undergo post-translational modifications (PTMs) after their initial biosynthesis to alter their structure and functions, enriching their complexity and functional diversity to regulate myriad cellular processes from signaling to gene expression.^1^ Among these PTMs is protein lipidation, which encompasses myristoylation, prenylation and palmitoylation.^2^

*S*-palmitoylation (often referred to more broadly as *S*-acylation) is a prominent modification that influences diverse cellular physiology and impacts around 10% of the human proteome, constituting receptors, enzymes, adhesion proteins, and more.^3–5^ Thus, its dysregulation is associated with broad pathologies, including cancers and immunological and neurodegenerative diseases.^3^ Understanding the mechanisms of this dynamic modification in normal and diseased conditions potentially offers substantial opportunities for developing targeted therapeutics.^6^

*S*-acylation is a reversible post-translational modification involving the addition of fatty acids of varying chain lengths (typically C14–C22, with C16 palmitate being the most prevalent)^8^ to cysteine residues of substrate proteins via a labile thioester linkage.^9^ A major subtype, *S*-palmitoylation, specifically refers to the conjugation of palmitate to cysteine thiols.^6^ It is named as such because palmitoylation occurs on the cysteine thiol, distinguishing it from *O*-acylation, which targets the serine/threonine hydroxyl group, and *N*-acylation, which modifies N-terminal residues.^2^ Mammalian *S*-acylation is catalyzed by palmitoyl *S*-acyltransferases (PATs), also known as ZDHHC enzymes that share 4–7 transmembrane domains (TMDs) and a conserved zinc-finger cysteine-rich region containing the catalytic Asp-His-His-Cys (DHHC) motif (Figure 1).^7^ The ZDHHC enzymes are primarily localized in Golgi apparatus and the endoplasmic reticulum (ER), while a small fraction is identified at the plasma membrane.^10^ Given that the ZDHHC enzymes can transfer a range of acyl chains, they are more accurately described as *S*-acyltransferases (*S*-ATs) than palmitoyl *S*-acyltransferases (PATs).^5^

**Figure 1:**
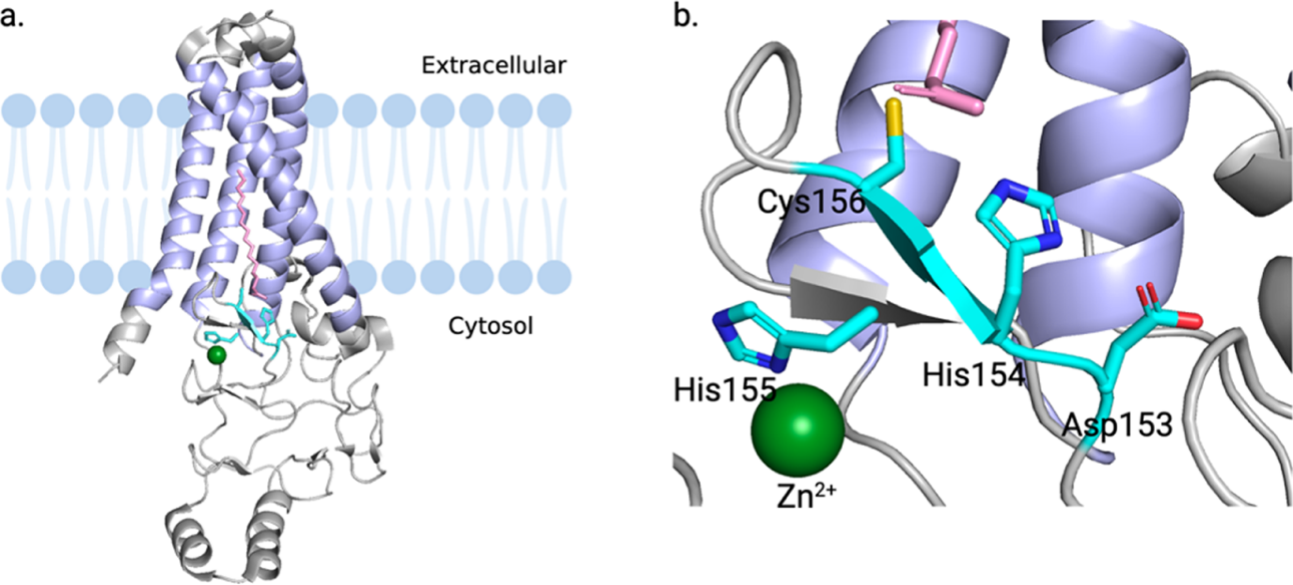
(a) Structure of human ZDHHC20 (PDB:6BML).^7^ Acyl-CoA (pink) inserts into the hydrophobic pocket created by the TMDs (lilac) and interacts with the ZDHHC motif (cyan). (b) Zoom-in of the ZDHHC motif. The zinc ion is represented as a green sphere, and the side chains as sticks, and colored by element (C-cyan, O-red, N-blue, S-yellow). Figure created using BioRender.

## Catalysis

### *S*-Acylation

The main ZDHHC enzyme with extensively established mechanistic insights is ZDHHC20 ^7^ based on structural analyses and many other experimental approaches. These include chemical biology combined with proteomics,^11^ cell-biology with fluorescence,^12^ and HPLC peptide-based assays.^13^ Crystal structures are also available for ZDHHC15 ^7^ and the ankyrin repeat region of ZDHHC17,^14^ while cryo-electron microscopy structures of ZDHHC9 in complex with an accessory protein GCP16 ^15^ and ZDHHC5 with the same partner^16^ have been solved very recently. However, we are still lacking detailed, and definitive, structural and mechanistic information for most enzymes in this category.

Hence, although 23 PATs have been identified in mammalian systems, it remains unclear whether all function as *S*-acyltransferases, and exact catalytic processes are not fully resolved. In the case of ZDHHC20, researchers postulated and confirmed a 2-step ping-pong mechanism, which involves initial enzyme autoacylation, followed by transfer of the acyl group to substrate proteins (Figure 2).^13^ In this model, palmitate-CoA inserts into the hydrophobic pocket formed by the TMDs, allowing PATs to autoacylate and form a palmitate-PAT intermediate, which catalyzes the slow step of the reaction: acyl chain transfer to the substrate protein.^6,11,12^

**Figure 2:**
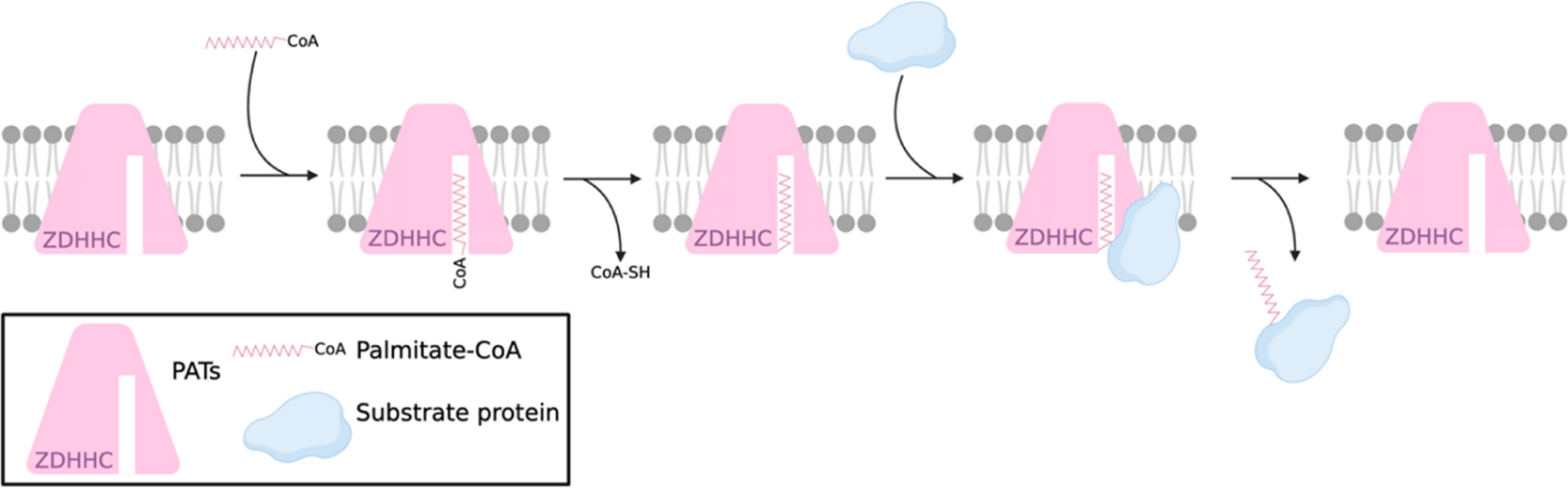
Overview of ping-pong *S*-palmitoylation model.^6^ Figure created using BioRender.

Despite the strong support for this ping-pong mechanism, it is uncertain whether it applies universally to all ZDHHCs. The detection of autoacylation is a key criterion for validating the ping-pong model. However, such intermediates have not been consistently observed across the entire ZDHHC family. Multiple experimental approaches, including using radio-labeled palmitate,^17^ alkyne functionalized fatty acids,^9^ fluorescent palmitoyl-CoA analogues,^18^ and azide-based probes,^9^ have been employed, yet comprehensive detection remains incomplete. Further investigations will determine whether autoacylation of some of the enzymes is incompatible with these detection techniques or if there is the possibility of noncanonical catalysis.^9,12,14,17,19^ Further complicating interpretation, inconsistent nomenclature across studies, including contradictory numbering of ZDHHC isoforms, hinders systematic comparison of these experimental findings.^12,17^ It has also been suggested that PATs could behave as intermediaries by forming a ZDHHC-palmitate-CoA substrate ternary complex, increasing the local palmitate-CoA concentration, facilitating direct palmitate-CoA attack on the substrate reactive cysteines.^20^ While PATs exhibit substrate specificity for palmitate-CoA, other structurally similar acyl groups can serve as substrates.^9^

Although many ZDHHC enzymes are likely to follow a ping–pong mechanism, the underlying chemical transformations of enzyme-catalyzed protein palmitoylation are not completely defined. Nonetheless, the conserved DHHC motif (Cys156, His154, Asp153) is central to catalysis and supports a general acid–base mechanism.^7^ During the autoacylation step, the aspartate 153 residue modulates the protonation state of the adjacent histidine, enabling it to act as a general base that deprotonates the catalytic cysteine to generate a reactive thiolate. This nucleophile attacks the carbonyl carbon of palmitoyl-CoA to yield a thioester-linked acyl–enzyme species at the active-site cysteine (e.g., Cys156; Scheme 1 ^15^). In the subsequent acyl transfer step, structural insights from ZDHHC20 indicate that His154 is positioned proximal to the thioester carbonyl and may function as a general acid, stabilizing the transition state by protonating the carbonyl oxygen and thereby increasing the electrophilicity of the acyl carbon. This facilitates nucleophilic attack by the substrate cysteine, resulting in transthioesterification and regeneration of the active DHHC motif.^7^ Moreover, the C-terminal cytoplasmic tail also contributes to enzymatic activity. Multiple ZDHHC isoforms (e.g., zDHHC5, 6, 8, 9, 16, 17 and 20) undergo *S*-acylation at conserved cysteine residues (Cys236/237/245), which is demonstrated to stabilize transmembrane domain architecture and enhance catalytic efficiency.^12^ Disruption of this modification diminishes autoacylation, highlighting its functional importance.^12^

**Scheme 1:**
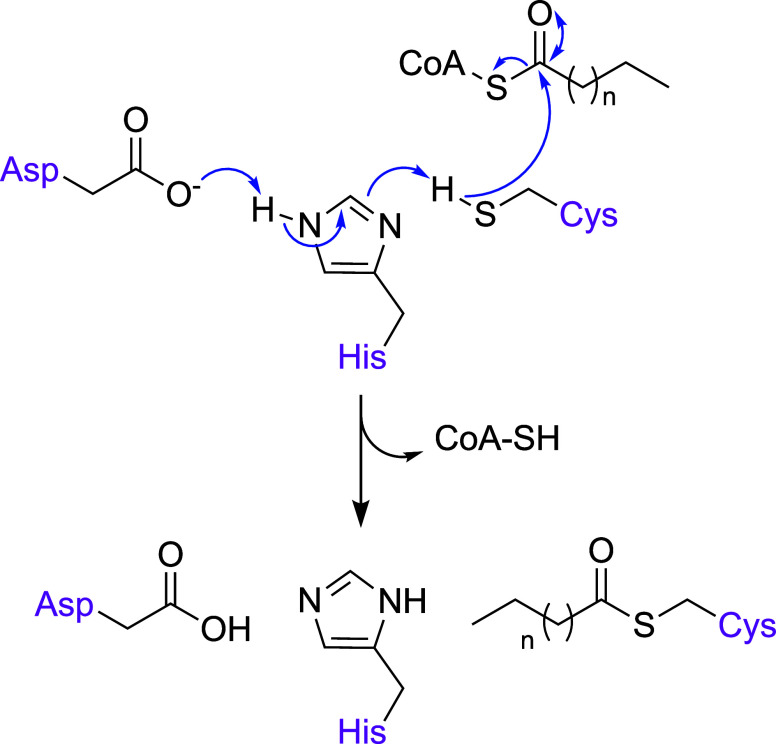
Mechanism of *S*-Palmitoylation

### Deacylation

*S*-acylation is a reversible posttranslational modification. Hence, acylated protein levels are closely coordinated via the dynamic interplay of acylation and deacylation. The enzymes responsible for *S*-deacylation primarily derive from the serine hydrolase superfamily and include acyl protein thioesterases (APTs), palmitoyl-protein thioesterases (PPTs), and α/β-hydrolase domain-containing 17 proteins (ABHD17s; Table 1).^2,6,21^ These enzymes share an active site serine, which cleaves the thioester bond.^6^ For clarity, we collectively refer to these enzymes as *S*-deacylases (*S*-DAs), in parallel with the *S*-acyltransferases (*S*-ATs).

**Table 1:**
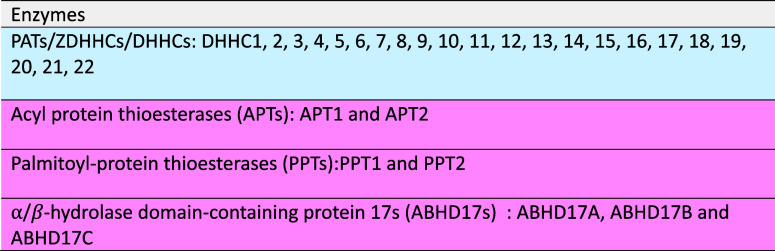
Summary of Mammalian Palmitoylation (Blue) and Depalmitoylation (Pink) Enzymes^5^ ^a^

a PATs, ZDHHCs, and DHHCs are used interchangeably in this review.

b See^3^ for a comprehensive account of all currently known biological targets and disease association.

#### Acyl-Protein Thioesterases

Most *S*-acylated proteins are deacylated by acyl protein thioesterases (APTs), with APT1 and APT2 also known as lysophospholipase 1­(LYPLA1) and lysophospholipase 2 (LYPLA2), respectively.^2,21^ Both enzymes possess dual lysophospholipase and thioesterase activities and share substantial sequence similarity (∼64%), including a conserved N-terminal cysteine residue (Cys2) that undergoes *S*-acylation and plays a critical role in membrane association.^23^

Despite their high homology, APT1 and APT2 display differential substrate selectivity. APT1 mediates agonist-dependent palmitoylation of β2-adrenergic receptors, whereas APT2 preferentially regulates substrates such as DHHC6.^2,22^ Nevertheless, both enzymes act on overlapping targets, including H-Ras, growth-associated protein-43 (GAP-43) and NMNAT2.^24^

Mechanistic studies of APT2 reveal a multistep membrane interaction process. APT2 first interacts with the membrane via long-range electrostatic attraction through a section of positively charged residues, allowing its hydrophobic β-tongue to anchor it. Subsequently, ZDHHC3/7 palmitoylates APT2, enhancing APT2 binding and membrane deformation to extract the target palmitate into its hydrophobic pocket, facilitating thioester bond hydrolysis near the catalytic site (Figure 3).^2,22^ APT1 can depalmitoylate itself and APT2, this regulation links to the fluid localization and depalmitoylation activities of APTs.^25^

**Figure 3:**
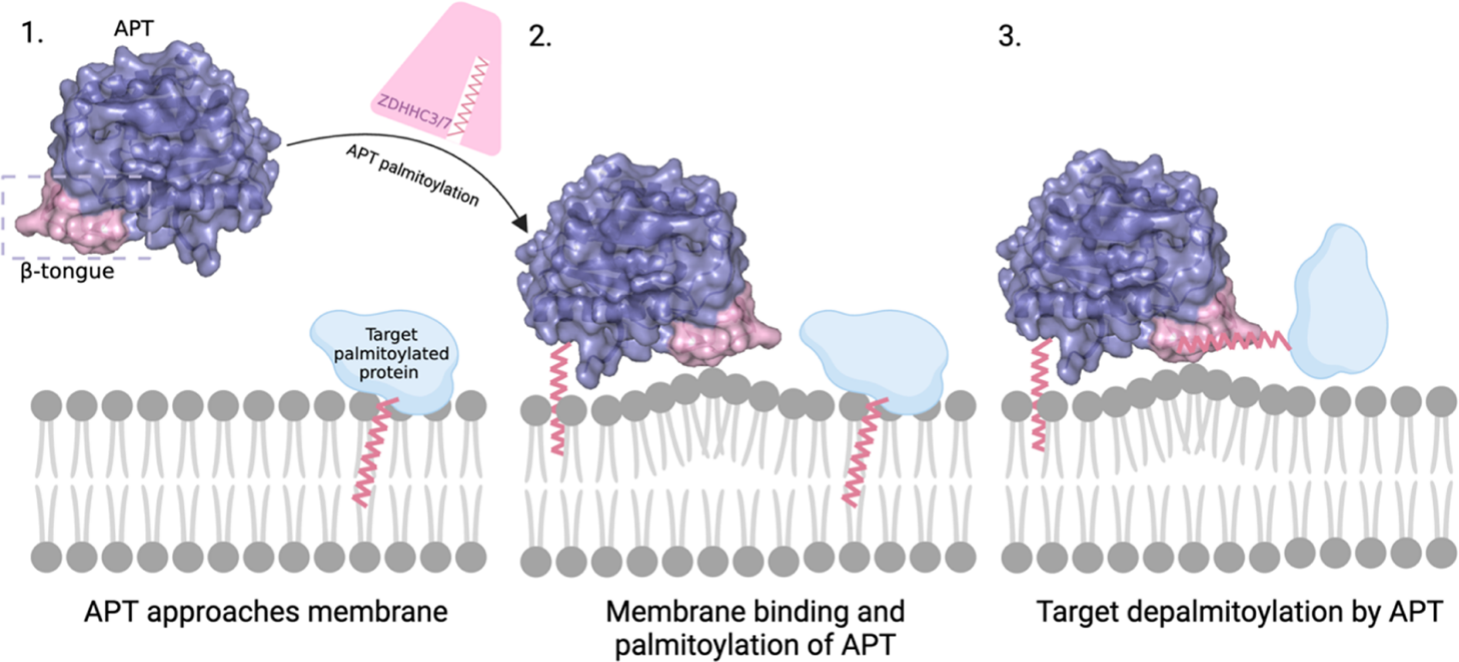
Deacylation by APT2 (PDB:6QGS).^22^ (1) APT2 approaches the membrane via long-range electrostatic attraction. (2) β-tongue insertion into the membrane. Acylation by ZDHHC3/7 anchors it into the membrane. (3) Once bound, it deforms the membrane and extracts palmitate for depalmitoylation.^22^ Figure created using BioRender.

#### Palmitoyl-Protein Thioesterases

Aside from APTs, protein palmitoyl thioesterase 1 (PPT1) deacylates proteins in lysosomes via the mannose 6-phosphate receptor-mediated pathway.^26^ Protein palmitoyl thioesterase 2 (PPT2) shares 26% sequence identity with PPT1 and preferentially hydrolyses palmitoyl-CoA over palmitoylated proteins.^27^ Despite their structural similarities, conformational differences in the lipid binding domain helices confer PPT2 substrate selectivity as its binding groove is smaller than PPT1, preventing its binding to fatty acids with bulkier headgroups like palmitoylcysteine, suggesting distinct, nonredundant functions.^28^ Nevertheless, the intricate interplay between the PPTs remains poorly characterized.

#### Alpha/Beta Hydrolase Domain-Containing Protein 17 A/B/C (ABHD17 A/B/C)

Within the ABHD family, although ABHD10 ^29^ and ABHD16A^30^ have been linked to deacylation, the ABHD17 subfamily^31^ represents the most extensively characterized deacylase. It comprises three isoforms, ABHD17A, ABHD17B, and ABHD17C, that are broadly expressed in vertebrates and localize to endosomal compartments, the plasma membrane (PM), as well as autophagosomes and lysosomes.^32^

Plasma membrane targeting of ABHD17 proteins is governed by their N-terminal region, which contains multiple conserved cysteine residues (C10, C11, C14, C15, C18), alongside a loop structure proximal to the N-terminus that flanks the hydrophobic cavity.^33^ Autoacylation of the N-terminal cysteine-rich domain promotes association with the PM, where most of the substrates are located.^34^ Mechanistic studies of ABHD17A support a stepwise trafficking pathway. Initial transport to the trans-Golgi network is driven by the hydrophobic and aromatic residues adjacent to the cysteine-rich motif. Subsequent palmitoylation at C14 and C15 occurs within this compartment, followed by sorting via a YXXØ motif to endosomal membranes, where additional palmitoylation at C10, C11 and C18 occurs and functions as a regulatory checkpoint that permits progression to the PM.^32^ Upon membrane delivery, insertion of the acylated N-terminus into the lipid bilayer, together with the engagement of the hydrophobic loop, stabilizes the enzyme and induces conformational rearrangement of the hydrophobic cavity, thereby enhancing substrate extraction and accommodation within the substrate-binding pocket.^31^ Moreover, disruption of this region, either by deletion of the N-terminus or mutation of key cysteine residues within the cysteine-rich motif, impairs membrane localization, while mutations within the hydrophobic loop alter pocket accessibility without preventing membrane association.^31,32^

Functionally, ABHD17 enzymes regulate the deacylation of substrates such as postsynaptic density protein 95 (PSD95),^33^ N-Ras^35^ and NOD2 ^36^ (an intracellular innate immune receptor), and additionally modulate ZDHHCs autopalmitoylation, underscoring the broader role in controlling acylation dynamics.

Overall, despite these insights, our detailed understanding of *S*-deacylases (*S*-DAs) and their structural features, substrate binding interactions and selectivity, and enzyme kinetics are still severely limited; more efforts are necessary to elucidate regulation of *S*-deacylation.

### Physiological Roles of *S*-Palmitoylation

The attachment of a fatty acid to proteins increases their hydrophobicity; consequently, subcellular localization, conformation, stability, and interactions with other molecules can be coordinated.

#### Subcellular Localization

The distinctive reversibility of *S*-palmitoylation enables it to mediate protein trafficking between organelles and localization to membranes. Acting as a lipid anchor, the hydrophobic palmitate enhances protein–membrane affinity.^6,37,38^ Among the proteins affected by *S*-palmitoylation, fatty acid translocase CD36 is a multifunctional membrane glycoprotein involved in fatty acid homeostasis and cellular inflammatory response, thereby synergistically contributing to nonalcohol steatohepatitis (NASH) development.^39^ Increased CD36 palmitoylation and localization to the hepatocyte PM were observed in mice with NASH, while CD36 inhibition reduced its hydrophobicity and PM localization, subsequently mitigating inflammatory responses and NASH development.^39^ Thus, CD36 palmitoylation can be targeted as a therapeutic strategy for NASH treatment.

#### Conformation and Stability

Beyond regulating protein localization, *S*-palmitoylation can alter protein conformation and stability. Caspase-6 (CASP6), a cysteine protease that coordinates neuronal degeneration during neuronal apoptosis, plays critical roles in neurological disorders like Alzheimer’s and Huntington’s diseases (AD and HD, respectively).^40^ Aberrant CASP6 activation is an early pathogenic event in HD, leading to huntingtin protein cleavage to generate toxic N-terminal HTT fragments, promoting neurodegeneration.^41^ DHHC17-mediated palmitoylation of CASP6 at Cys264 and Cys277 was predicted using computational modeling to inhibit CASP6 activation via two mechanisms: First, palmitoylation at the first site obstructs the activation site and lengthens the catalytic duo (Cys163/His121) distance, preventing Cys163 nucleophilic attack on Asp.^41^ Second, Cys277 palmitoylation introduces steric hindrance of the long palmitoyl chain, intercepting the approach of another CASP6 molecule and subsequent dimerization and activation.^41^

Programmed death ligand 1 (PD-L1) is highly expressed in cancer cells and interacts with programmed cell death protein 1 (PD-1) on T-cells to impede T-cell antitumor activity.^42,43^ PD-L1 palmitoylation at Cys272 by DHHC3 increases its stability by arresting its ubiquitination, whereas inhibiting palmitoylation supports its lysosomal degradation by inducing ubiquitination.^43^ Thus, these discoveries propose novel approaches to conquering PD-L1-mediated immune evasion in cancers.

These examples demonstrate how *S*-palmitoylation critically influences protein conformation and stability, underscoring its importance in pathophysiological processes.

#### Biomolecular Interactions

Finally, *S*-palmitoylation-induced lipophilicity can alter protein–protein interaction. Human adhesion G-protein coupled receptors (aGPCRs) are critical molecular switches that regulate multitudinous processes; orphan receptor GPR97 is a member of the aGPCR family that couples with the heterotrimeric G_0_ protein to initiate downstream signaling, modulating lymphatic remodelling and B-cell development.^44,45^ Palmitoylation at the cytosolic tail of the G_0_ protein facilitates the acyl chain insertion, 17 Å deep into the seven TM core of the GPR97-G_0_ complex, resulting in allosteric stabilization of residues within the ligand binding pocket of GPR97, strengthening its affinity to ligands like glucocorticoids.^44^

### *S*-Palmitoylation in Disease States

#### Cancer

The relationship between *S*-palmitoylation and many cancers is becoming progressively elucidated, and its tumor or antitumor effects vary significantly with each cancer.^46^

Several tumor-associated proteins require *S*-palmitoylation for localization onto the PM to activate oncogenic pathways. Namely, δ-catenin is an oncogenic protein upregulated in prostate cancer.^47^ It interacts with epidermal growth factor receptor protein (EGFR) on the PM by preventing its ubiquitination, activating the extracellular signal-regulated kinase (ERK1/2) pathway, and resulting in increased cancer proliferation and metastasis.^47,48^ It also interacts with PM E-cadherin, a protein responsible for cell adhesion and tumor suppression, to induce its degradation, releasing β-catenin and carcinogenic signals.^49^ Mutation of δ-catenin Cys-palmitoylation sites to serine, forming a “depalmitoylated” δ-catenin mutant, reduced its localization to the PM, decreasing its interactions with both EGFR and E-cadherin proteins.^48^ Thus, prostate cells with mutant δ-catenin had lower cell proliferation, migration, and adhesion.^48^

Similarly, disrupting ZDHHC9 expression or impairing PD-L1 palmitoylation through site-specific point mutation in breast cancer cells enhances responsiveness to T-cell activity, thus attenuating tumor growth.^50^ Conversely, the ZDHHC22-mediated palmitoylation of the mammalian target of rapamycin (mTOR) protein induces its destabilization, inhibiting the subsequent phosphoinositide three kinases (PI3)/AKT/mTOR pathway, leading to reduced breast cancer tumor growth and proliferation through cell cycle arrest and apoptosis.^51^ Thus, *S*-palmitoylation plays disparate roles within the carcinogenesis of the same cancer.

*S*-palmitoylation can suppress tumor growth by activating tumor suppressor signaling pathways, and the overexpression of ZDHHCs is associated with cancer inhibition. Insulin-like growth factor 2 mRNA binding protein (IGF2BP1) regulates RNA stability and translation.^52^ Palmitoylation by ZDHHC1 at Cys^337^ enables IGF2BP1 to bind to the N^6^-methyladenosine (m6A) site of its downstream target lipase-G (LIPG) mRNA, destabilizing LIPG mRNA via m6A modification, subsequently reducing LIPG expression.^52^ LIPG, an endothelial lipase that coordinates lipid metabolism, is linked to the aberrant energy provision in cancer.^52^ Thus, its downregulation impedes intracellular lipid high-density lipoprotein (HDL) uptake in colorectal cancer, reducing cell proliferation.^52^

Overall, *S*-palmitoylation contributes significantly and distinctively to carcinogenesis, striking a balance between an oncoprotein and tumor suppressor behavior.^46^

#### Immunity and Autoimmune Diseases

*S*-palmitoylation is implicated in numerous immune signaling pathways like the stimulator of interferon gene (STING), phagocytosis, and apoptosis.^53^ Therefore, its aberrant activity contributes to the development of various inflammatory and autoimmune diseases, including colitis and inflammatory bowel diseases (IBD).^54,55^

Cytosolic DNA indicates pathogen invasion, cellular stress, and tissue damage; therefore, detecting cytosolic DNA is vital for innate immunity. Cyclic GMP-AMP synthase (cGAS) protein is the most prominent cytosolic DNA detector: it identifies and binds onto cytosolic double-stranded DNA,^56^ followed by dimerization into a 2:2 cGAS-DNA complex to activate the STING pathway, triggering subsequent synthesis of immune mediators like antiviral type 1 interferons (IFNs) and proinflammatory cytokines, conferring defense against DNA viruses;^56^ cGAS cannot discriminate between self-and foreign DNA.^57^ Therefore, cytosolic self-DNA presence, including mtDNA and chromatin, during mitosis necessitates meticulous control of the cGAS-STING pathway to prevent overactivation and chronic inflammation.^57^ *S*-palmitoylation of cGAS at Cys474 results in conformational changes that decrease DNA binding, thereby limiting cGAS-DNA complex formation and subsequent pathway activation.^58^ Additionally, cGAS-DNA binding leads to increased cGAS colocalization with ZDHHC18 and reduced binding free energy of cGAS and ZDHHC18.^58^ Thus, Shi et al. proposed that this modification serves as a negative feedback of the cGAS-STING pathway to fine-tune the cGAS-STING-mediated immune response.^58^ Interestingly, within the same pathway, STING is *S*-palmitoylated at Cys88/91, allowing it to cluster within lipid rafts of the Golgi apparatus, to recruit TANK-binding kinase 1 (TBK1) and interferon regulatory factor 3 (IRF3) and trigger IRF3 phosphorylation to initiate transcription of immunity genes.^59^ Thus, *S*-palmitoylation has multifaceted influences within a single pathway (Figure 4a).

**Figure 4:**
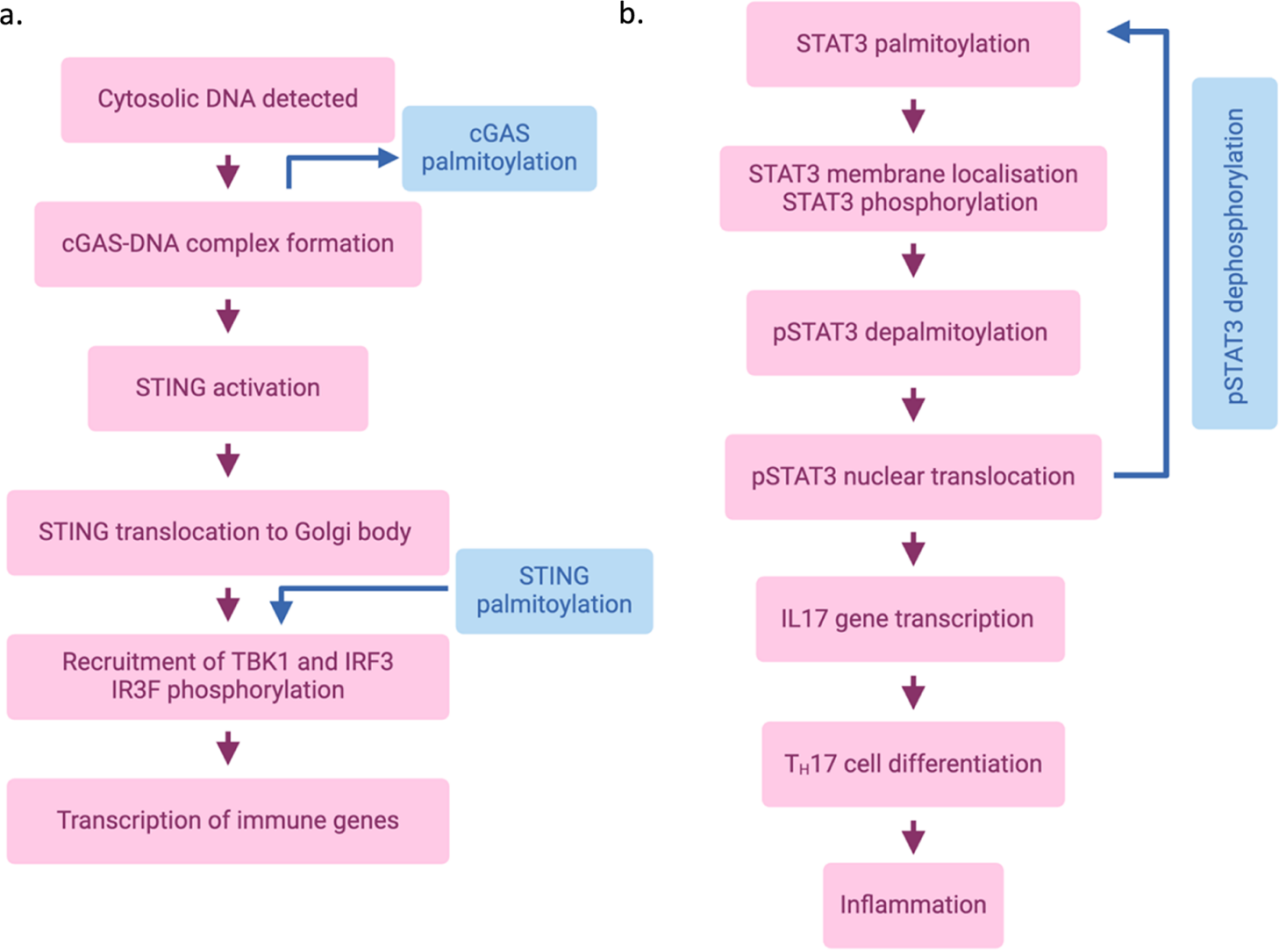
(a) The role of *S*-palmitoylation in the cGAS-STING pathway.^56^ (b) The role of *S*-palmitoylation in STAT3 activation.^53^ Figure created using BioRender.

Beyond innate immunity, *S*-palmitoylation plays a critical role in adaptive immunity. T-helper 17 (T_H_17) cells regulate tissue homeostasis, particularly in the intestinal mucosa. Its overactivation and differentiation are involved in IBD and colitis.^54,60^ T_H_17 cell differentiation is triggered by the palmitoylation-depalmitoylation cycle of signal transducer and activator of transcription gene 3 (STAT3), mediated by DHHC7 and APT2. First, STAT3 is palmitoylated by DHHC7 at Cys108 to induce membrane localization and phosphorylation.^54^ Phosphorylated STAT3 (p-STAT3) is then depalmitoylated by APT2, translocating it to the nucleus, activating genes to trigger T_H_17 cell differentiation (Figure 4b).^54^ Thus, targeting the regulation of STAT3 palmitoylation to suppress T_H_17 cells offers a promising avenue in treating autoimmune disorders caused by atypical T_H_17 activity.

Collectively, these insights emphasize the importance of *S*-palmitoylation in regulating immunity and highlight a potential target for tackling inflammatory and autoimmune disorders.

#### Neurodegenerative Diseases

*S*-palmitoylation is vital for healthy brain function, from neuronal dendrite growth and neurotransmission to controlling protein transport between synaptic membrane and intracellular organelles for synaptic plasticity.^37,61,62^ Thus, dysregulated *S*-palmitoylation is associated with varying neurodegenerative disorders, including AD, HD, and more.^37,61^ This section will utilize AD to examine how *S*-palmitoylation drives the pathogenesis of neurological disorders.

AD is characterized by neurotoxic extracellular amyloid-beta (Aβ) plaques and intracellular neurofibrillary tangles (NFTs), manifesting as cognitive decline and progressive memory loss.^63^ Aβ is produced through the proteolytic processing of amyloid precursor protein (APP) by β- and γ-secretases, both of which are coordinated by *S*-palmitoylation.^62,63^ Namely, palmitoylation of β-site APP cleaving enzyme 1 (BACE1) at Cys474, 478, 482, and 485 reduced Aβ plaques, aggregation and attenuated memory loss in AD mice models.^64^ ZDHHC12-mediated palmitoylation of α-secretase facilitates nonamyloidogenic α-cleavage of APP and protects against Aβ production.^65^ Conversely, palmitoylation of APP at Cys186 &187 increases its interaction with the lipid rafts, inducing γ-secretase-mediated cleavage and Aβ production.^66^

Selenoprotein K (SELENOK) is a critical protein regulating brain immune responses via the microglia.^67^ Specifically, SELENOK modulates the palmitoylation of its downstream target: CD36 protein, a microglial plasma membrane receptor essential for Aβ recognition, binding, and clearance via microglial phagocytosis.^67^ Through interaction with ER-localized DHHC6, SELENOK promotes CD36 palmitoylation and localization to the microglial PM, facilitating Aβ phagocytosis.^67^ Thus, downregulated SELENOK and impaired CD36 palmitoylation are observed in AD mouse models, likely due to inhibited microglial Aβ phagocytosis exacerbating cognitive deficits in mice.^67^

The influence of *S*-palmitoylation extends to other neurological disorders. Mutant huntingtin protein exhibits reduced palmitoylation while APT inhibition exerts neuroprotective effects in HD.^68^ In PD, synaptotagmin-11 palmitoylation at Cys39 and 40 is correlated to elevated α-synuclein binding to membranes and accumulation within neurons,^69^ and APT1 inhibition promotes healthy microtubule-associated protein (MAP6) interaction with vesicles, decreasing neurotoxicity.^69^

These examples emphasize *S*-palmitoylation as a critical contributor to neuronal health, exerting complex and diverse effects on brain immune responses, protein processing, and trafficking.

#### Challenges and Outlook

Despite its discovery in 1979,^70^ many aspects of *S*-acylation remain unresolved; information about the enzyme kinetics and substrate recognition of palmitoylation enzymes is lacking. Additionally, our understanding of *S*-acylation regulatory mechanisms, alongside its interplay with other PTMs, is still rudimentary. Therefore, utilizing *S*-acylation as a therapy target remains challenging.

Addressing these challenges is complicated by the lack of PAT selective inhibitors. 2-Bromopalmitic acid (2-BP), a common competitive irreversible inhibitor, blocks palmitoylation by preventing palmitate-PAT intermediate formation (Figure 5).^71^ However, its significant off-target inhibition on APTs and other cysteine-containing enzymes, like glucose-6-phosphatase and monotriacylglycerol transferases,^71^ alongside its cytotoxicity, limits its therapeutic potential and complicates the interpretation of *S*-palmitoylation kinetics involving 2-BP.^71,72^ Other inhibitors, including cerulenin and tunicamycin, suffer similarly from poor selectivity and toxicity.^73^ In comparison, cyano-myracrylamide (CMA) looks more promising. It has been demonstrated to possess similar potency, lower toxicity and higher selectivity without inhibiting *S*-deacylase.^74^ However, it is nonspecific, targeting a wide range of ZDHHCs which limits its usefulness. Ergo, there is an urgent need to develop more selective PAT inhibitors.

**Figure 5:**
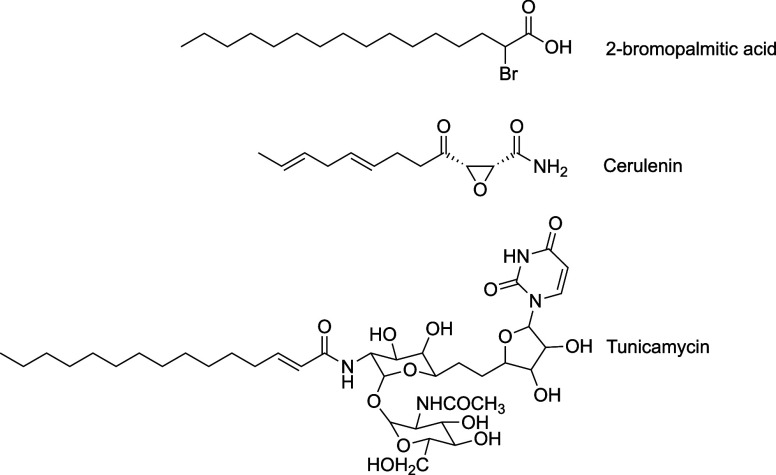
PAT inhibitors.

Other modern drug discovery routes that influence *S*-palmitoylation are beginning to emerge including peptide design^75^ and targeted protein degradation.^76^

Improvements in detection methodologies have advanced the field. Early approaches relied on the laborious and low-sensitivity incorporation of radiolabeled fatty acids into proteins, followed by immunoprecipitation.^6,77^ The revolutionary acyl-biotin exchange (ABE) method, introduced in 2004, coupled with LC–MS proteomics, enabled the global profiling of palmitoylated proteins.^6,77^ This technique involves first blocking the free thiols of palmitoylated protein using *N*-ethylmaleimide (NEM), followed by selective cleavage of the palmitate-thioester group with hydroxylamine, allowing palmitoylation sites to be labeled and detected (Figure 6).^6,77^ Other acyl-exchange methods like acyl-resin assisted capture (acyl-RAC)^78^ and acyl-polyethylene-glycol exchange (acyl-PEG)^79^ are also increasingly utilized (Figure 6).^6^ However, ABE and acyl-RAC can exhibit false positives as they merely confirm thioester bond presence.^80^ Additionally, they cannot illustrate palmitoylation-depalmitoylation dynamics or palmitoylation stoichiometry.^80^ Acyl-PEG improves on this by labeling released cysteines with PEG, inducing mass shift on SDS-PAGE dependent on the number of palmitoylation sites and palmitoylation stoichiometry.^80^

**Figure 6:**
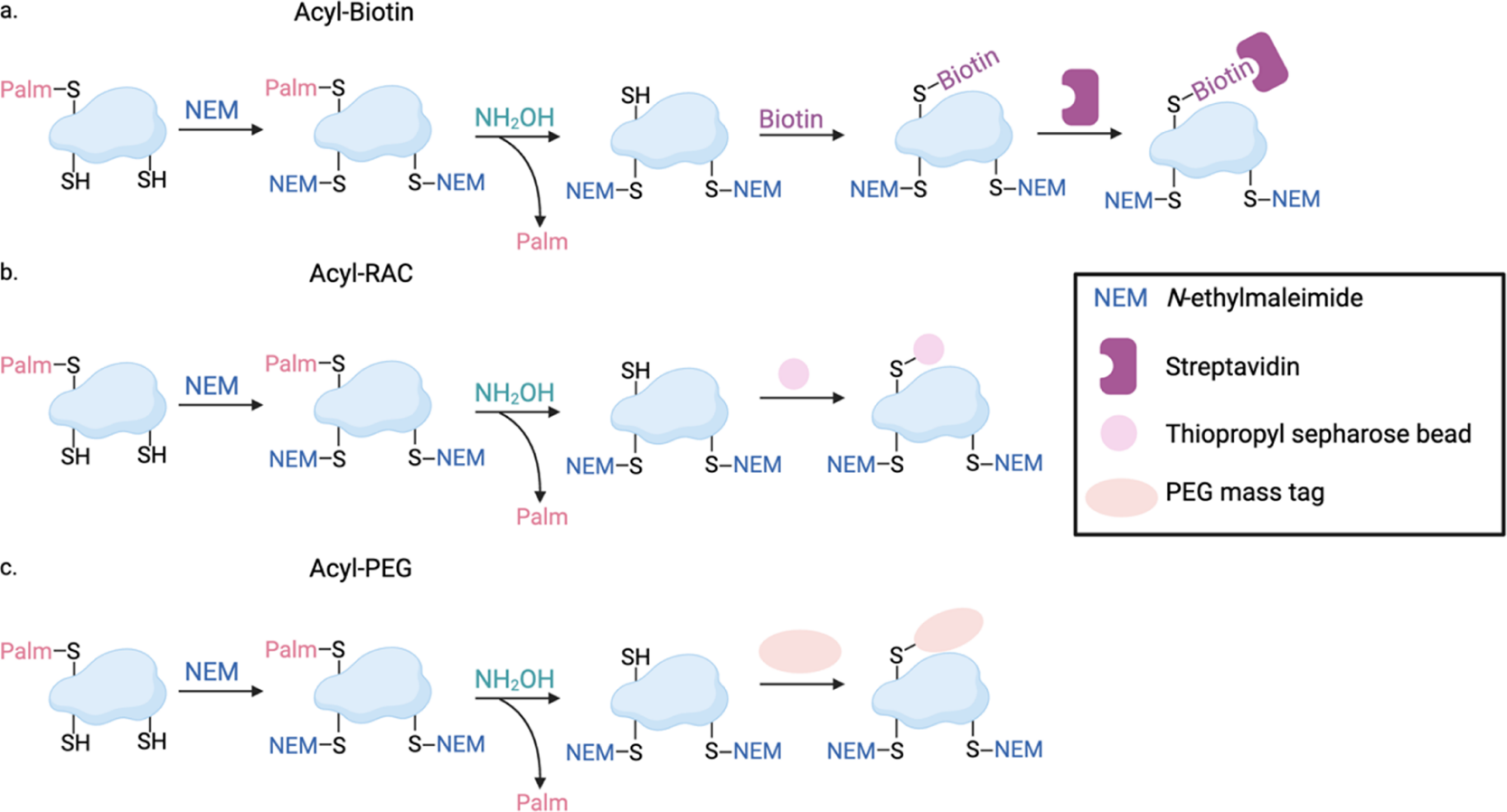
Acyl-exchange methods. (a) Acyl-biotin exchange (ABE): free thiols are blocked with NME, followed by hydroxylamine-mediated cleavage of thioester and biotin labeling for streptavidin capture. (b) Acyl-RAC: after NME blocking and hydroxylamine treatment, exposed cysteines are captured by thiopropyl sepharose resin. (c) Acyl-PEG: NME blocking and hydroxylamine cleavage enable labeling with PEG mass tag. Figure created using BioRender.

Alternatively, biorthogonal chemistry provides new investigative tools for *S*-palmitoylation with lower false positive rates: alkyne- or azide-modified fatty acid analogues are integrated into proteins, enabling *S*-palmitoylation detection with Staudinger ligation or Click reaction (Figure 7).^9,81,82^ While copper toxicity in click chemistry limits live-cell imaging, combining it with a “bump-hole” chemical-genetic system enables PAT-specific labeling and new PAT target identification, offering advantages over acyl-exchange methods.^11^ Diverse azido, alkynyl, and prenyl probes,^77,83^ designed for biorthogonal detection, show varying success; despite their limitations, 2-BP, cerulenin, and their analogues serve as valuable probes.^84^ Considering the drawbacks discussed, synergising techniques is recommended for better *S*-palmitoylation characterization.

**Figure 7:**
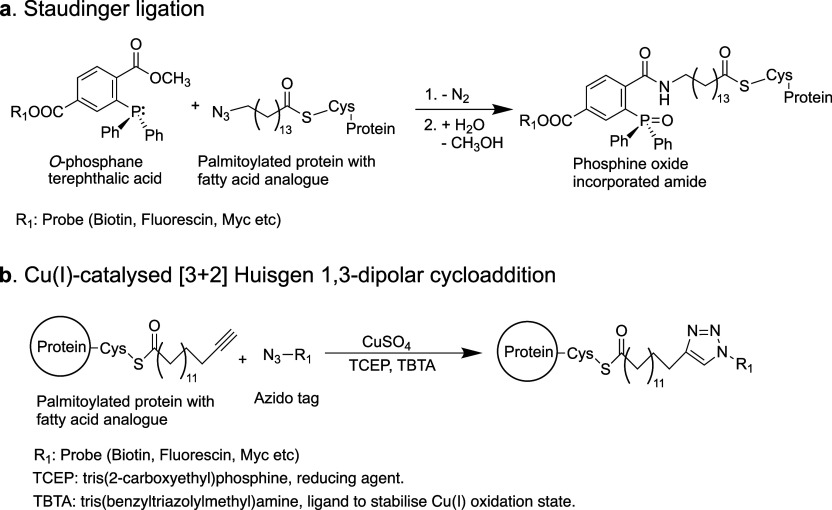
(a) Detection of palmitoylated protein with fatty acid analogue (azido-palmitoyl-CoA).^81^ (b). Detection of palmitoylated protein with ω-alkynyl-palmitate analogue by click chemistry.^82^

Alongside detection tools, computational systems and experimental methodologies provide insights into the *S*-acylation network. Deep-learning models predict acylation sites, while orthogonal design strategies identify novel targets, offering complementary approaches to understanding *S*-acylation.^4,85–87^

Overall, *S*-palmitoylation is a critical PTM that significantly impacts protein localization, conformation, and bimolecular interactions, influencing diverse pathways and pathologies. As proteomics technologies improve, addressing existing challenges becomes increasingly feasible, driving significant discoveries. Finally, integrating disease-specific proteomics data with enzyme interaction and expression data further demystifies *S*-palmitoylation and highlights promising therapeutic targets.
